# Neurodevelopmental Consequences of Sub-Clinical Carbon Monoxide Exposure in Newborn Mice

**DOI:** 10.1371/journal.pone.0032029

**Published:** 2012-02-10

**Authors:** Ying Cheng, Adia Thomas, Feras Mardini, Shannon L. Bianchi, Junxia X. Tang, Jun Peng, Huafeng Wei, Maryellen F. Eckenhoff, Roderic G. Eckenhoff, Richard J. Levy

**Affiliations:** 1 Division of Anesthesiology and Pain Medicine, Children's National Medical Center, The George Washington University School of Medicine and Health Sciences, Washington, D.C., United States of America; 2 Department of Anesthesiology and Critical Care, University of Pennsylvania School of Medicine, Philadelphia, Pennsylvania, United States of America; King's College London, United Kingdom

## Abstract

Carbon monoxide (CO) exposure at high concentrations results in overt neurotoxicity. Exposure to low CO concentrations occurs commonly yet is usually sub-clinical. Infants are uniquely vulnerable to a variety of toxins, however, the effects of postnatal sub-clinical CO exposure on the developing brain are unknown. Apoptosis occurs normally within the brain during development and is critical for synaptogenesis. Here we demonstrate that brief, postnatal sub-clinical CO exposure inhibits developmental neuroapoptosis resulting in impaired learning, memory, and social behavior. Three hour exposure to 5 ppm or 100 ppm CO impaired cytochrome c release, caspase-3 activation, and apoptosis in neocortex and hippocampus of 10 day old CD-1 mice. CO increased NeuN protein, neuronal numbers, and resulted in megalencephaly. CO-exposed mice demonstrated impaired memory and learning and reduced socialization following exposure. Thus, CO-mediated inhibition of neuroapoptosis might represent an important etiology of acquired neurocognitive impairment and behavioral disorders in children.

## Introduction

Carbon monoxide (CO) is a colorless and odorless gas generated primarily as a by-product of incomplete combustion of hydrocarbons [1]. When inspired, CO diffuses across the alveolar capillary membrane through plasma and binds to hemoglobin to form carboxyhemoglobin (COHb) [2]. Because the affinity of hemoglobin for CO is 240 times greater than that for oxygen, high COHb levels can impair tissue oxygen delivery by interfering with oxygen binding to and dissociation from hemoglobin [3], [4]. Overt CO toxicity results from tissue hypoxia when COHb levels are greater than 70% and signs and symptoms first appear when COHb is greater than 10% [4], [5]. Acute exposure to CO levels greater than 800 parts per million (ppm) can rapidly cause brain injury, cerebral edema, coma, and death [6]. Brief exposure to 220 ppm CO results in headache, dizziness, and impaired judgment while exposure to less than 120 ppm CO is usually sub-clinical [6]. Infants are more susceptible to the toxic effects of CO than adults due to higher metabolic rates and the presence of fetal hemoglobin, which binds CO more avidly than adult hemoglobin [1]. Prenatal CO exposure has been shown to result in decreased birth weight, small infant head circumference, behavioral abnormalities, and disruption in cognitive function while chronic low concentration postnatal CO exposure has been shown to impair the developing rat auditory system [7], [8], [9]. The effects of brief postnatal sub-clinical CO exposure on the developing brain, however, are unknown.

Programmed cell death is a widespread phenomenon that occurs within the central nervous system coincident with proliferation, migration, and differentiation [10]. Developmental neuroapoptosis occurs in two waves within the brain: during mid-embryogenesis (after gestational day 7 in the mouse) and in the early postnatal period (first few weeks of life in rodents) [11]. The early wave is important for progenitor cell size, morphogenesis, proliferation, and differentiation, while the postnatal wave is critical for synaptogenesis, removal of aberrant neuronal connections, and matching input size with the target field [10]. Thus, the early wave regulates the pool size while the later wave ensures proper wiring [12]. Apoptosis occurs in approximately 2% of cells in the developing cortex at any time during neurogenesis and in 24–30% of all cortical neurons in the postnatal period [13], [14]. Any insult that interferes with or interrupts the process of programmed cell death in the developing brain could have dramatic and long lasting implications with regard to neurocognitive development. Here we demonstrate that brief, postnatal sub-clinical CO exposure inhibits developmental neuroapoptosis by impairing cytochrome c release from mitochondria, resulting in impaired learning and memory and social behavior. These results indicate that CO-mediated inhibition of neuroapoptosis might represent an important etiology of acquired neurocognitive impairment in children and could have important public health consequences.

## Results

### Sub-clinical CO exposure in newborn mouse pups

To investigate the effects of sub-clinical CO on developmental neuroapoptosis, we exposed 10 day old CD-1 mouse pups to 0 ppm CO (air), 5 ppm CO (low concentration sub-clinical exposure), or 100 ppm CO (upper limit of sub-clinical exposure) for 3 hours. Postnatal day 10 (PND 10) was chosen because synaptogenesis peaks on day 7 in rodents and is completed by the second or third week of life [11], [14]. Although extrapolating the timing of brain development from animal models to humans is inexact, many experts agree that PND 4–9 in rodents is equivalent to the third trimester in humans while others believe that rodent neurodevelopment at PND 7 equates to the human brain at term birth [15], [16]. Thus, PND 10 in the mouse is probably analogous to a postnatal time point within human infancy [15], [16], [17]. Three hour CO exposure was chosen to represent a brief exposure as can occur in a variety of environments (re-breathing exhaled CO during general anesthesia, exposure to vehicle exhaust in heavy traffic or parking garages and tunnels, etc.) (http://www.epa.gov/airquality/carbonmonoxide/) [18].

**Figure 1:**
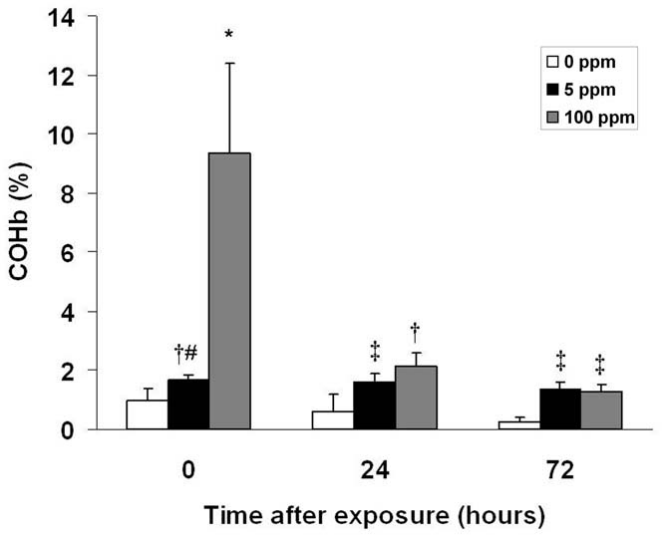
Carboxyhemoglobin (COHb) levels following CO exposure. COHb levels were measured immediately following 3-hour exposure (0 hours after exposure), 24 hours post exposure, and 72 hours post exposure in separate cohorts. Values are expressed as percentage (%) COHb means plus standard deviation. N = 8 animals per cohort. *P<.001 vs. all cohorts. **†** *P*<.05 vs. all 0 ppm cohorts. **‡** *P*<.05 vs. 0 ppm 24, 72 hrs. **#** *P*<.05 vs. 100 ppm 72 hrs.

COHb levels significantly increased immediately following exposure in both CO exposed cohorts, approximating levels expected in humans following a similar time-weighted exposure (figure 1) [6]. Importantly, resultant COHb levels were below the threshold that are known to elicit signs and symptoms in humans and were markedly less than values known to result in tissue hypoxia [4], [5]. Thus, 3-hour exposure to 5 ppm or 100 ppm CO in 10 day old mouse pups represents a sub-clinical exposure. COHb levels returned to PND 10 air-exposed control values 24 hours following 5 ppm CO exposure and 72 hours following 100 ppm CO exposure (figure 1). Importantly, COHb levels were significantly higher in both CO exposed cohorts compared to time-matched control values on PND 11 and PND 13 (24 and 72 hours post exposure, respectively) (figure 1).

### Brief sub-clinical CO exposure inhibits developmental neuroapoptosis

**Figure 2:**
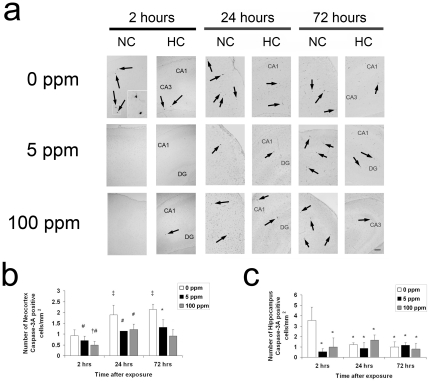
Activated caspase-3 decreases following CO exposure. Immunohistochemistry for activated caspase-3 was performed on coronal sections 2 hours (hrs), 24 hrs, and 72 hrs post exposure. (**a**) Representative sections imaged at 10× from somatosensory neocortex (NC) and hippocampus (HC) obtained at the 2-hour time point, 24 and 72 hours following 3-hour exposure to air (0 ppm CO), 5 ppm CO, or 100 ppm CO are depicted. Activated caspase-3 positive neurons undergoing degeneration within the boxed in region in layer IV of the somatosensory neocortex are magnified in the inset of the 0 ppm CO section 2 hours post exposure. Black arrows indicate activated caspase-3 stained cells. CA1, CA3, dentate gyrus (DG) regions of HC are labeled. Scale bars, 100 µm. Quantification of activated caspase-3 stained cells in (**b**) neocortex and (**c**) hippocampus are demonstrated. Values are expressed as means plus standard deviation. (**b**) *P<.05 vs. 0 ppm 72 hrs. **‡** *P*<.05 vs. 0 ppm 2 hrs. **#** *P*<.05 vs. 0 ppm 24, 72 hrs. **†** *P*<.05 vs. 5 ppm 24, 72 hrs. (**c**) *P<.02 vs. 0 ppm 2 hrs. N = 3–4 animals per cohort.

**Figure 3:**
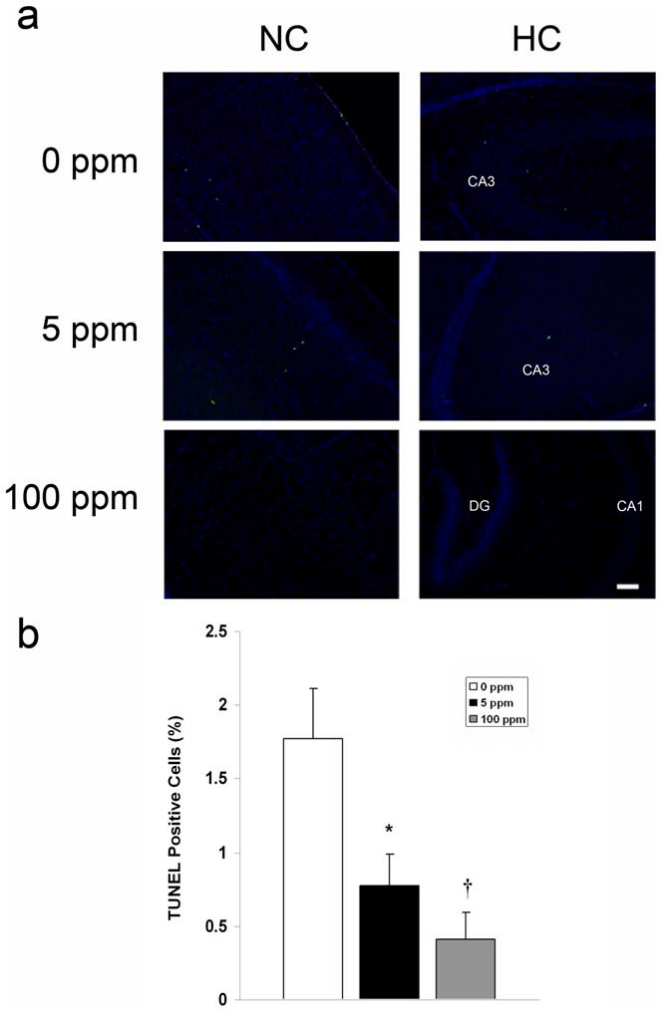
Neuroapoptosis decreases following CO exposure. TUNEL assays were performed on coronal sections 2 hours post exposure. (**a**) Representative sections imaged at 10× from somatosensory neocortex (NC) and hippocampus (HC) 2 hours following 3-hour exposure to air (0 ppm CO), 5 ppm CO, or 100 ppm CO are depicted. Green TUNEL positive nuclei are visible. CA1, CA3, dentate gyrus (DG) regions of HC are labeled. Scale bars, 100 µm. (**b**) Quantification of total TUNEL positive nuclei from NC and HC in 3–4 non-serial coronal sections is demonstrated. Values are expressed as means plus standard deviation. N = 3–4 animals per cohort. *P<.02 vs. 0 ppm. **†** *P*<.01 vs. 0 ppm.

**Figure 4:**
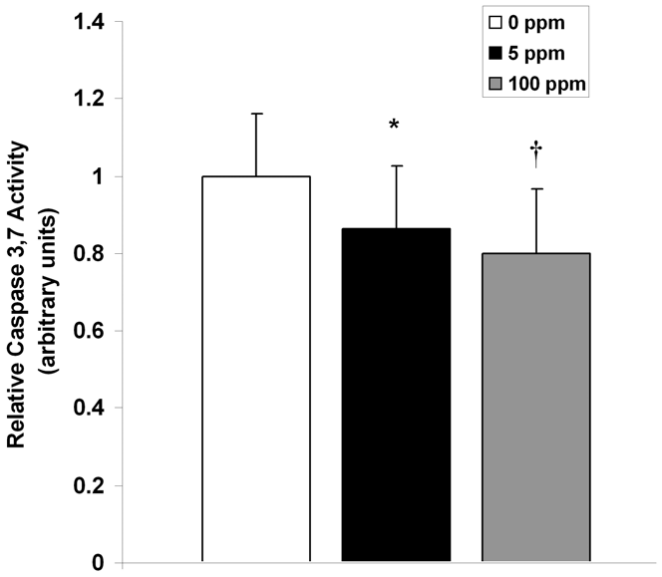
Caspase-3 activity decreases following CO exposure. Relative brain caspase-3,7 activity was measured in brain 2 hours post exposure. Values are expressed as means plus standard deviation. N = 10 animals per cohort. *P<.04 vs. 0 ppm. **†** *P*<.01 vs. 0 ppm.

The postnatal wave of programmed cell death in the developing brain is mediated by the intrinsic pathway of apoptosis [14]. Cell death follows an ascending gradient in rodents from subcortical regions to upper cortical regions with the majority of apoptosis occurring in the somatosensory cortex, medial cortical regions, the cortical subplate, and hippocampus in the first 10 postnatal days [19]–[21]. Thus, we determined the effect of CO on neuroapoptosis in the somatosensory neocortex and hippocampus by assessing for activated caspase-3 with immunohistochemistry and terminal deoxynucleotidyl transferase (TdT)-mediated UTP nick end-labeling (TUNEL) staining on slide mounted brain sections and by measuring brain caspase-3,7 activity. Pups were evaluated for up to 72 hours following a 3-hour exposure to either 0 ppm CO (air), 5 ppm CO, or 100 ppm CO on PND 10. Variation in temporal progression of developmental apoptosis was seen in different brain regions in air-exposed controls (figure 2). The number of activated caspase-3 positive cells increased from PND 10 to PND 13 in the neocortex while the number of activated caspase-3 positive cells in the hippocampus of air-exposed controls decreased over time (figure 2). This is consistent the temporal variation of neuronal apoptosis and synaptogenesis in different regions of the developing brain [11]. The majority of activated caspase-3 positive cells were localized to layers II, IV, and V of the neocortex and CA1 and CA3 of the hippocampus. CO exposure resulted in significantly less activated caspase-3 positive cells in the neocortex 24 and 72 hours post exposure compared to time-matched air-exposed controls (figure 2). In the hippocampus, CO exposure significantly decreased the number of activated caspase-3 cells 2 hours following exposure (figure 2). Consistent with these findings, caspase-3,7 activity and the number of TUNEL positive nuclei decreased significantly following CO exposure in a dose dependent manner (figures 3, 4).

### Sub-clinical CO exposure impairs cytochrome c release

**Figure 5:**
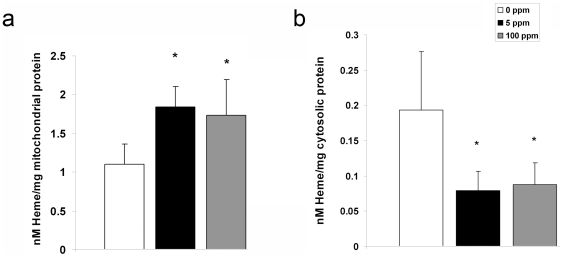
Cytochrome c release is impaired following CO exposure. Heme c content within (**a**) mitochondria and (**b**) cytosol is demonstrated. Values are expressed as means plus standard deviation. *P<.05 vs. 0 ppm. N = 8 animals per cohort.

Release of cytochrome c from the mitochondrial intermembrane space into cytosol is one of the most upstream events to initiate the intrinsic apoptosis pathway [14]. This results in apoptosome formation, subsequent caspase-3 activation, and DNA fragmentation [14]. To assess the effect of CO exposure on cytochrome c release in the developing brain, we measured the amount of heme c (the heme moiety of cytochrome c) in brain mitochondrial and cytosolic fractions using spectrophotometry. Mitochondrial levels of heme c were significantly higher while cytosolic levels were significantly lower in the brain following CO exposure compared to air-exposed controls, indicating CO-mediated inhibition of cytochrome c release (figure 5).

**Figure 6:**
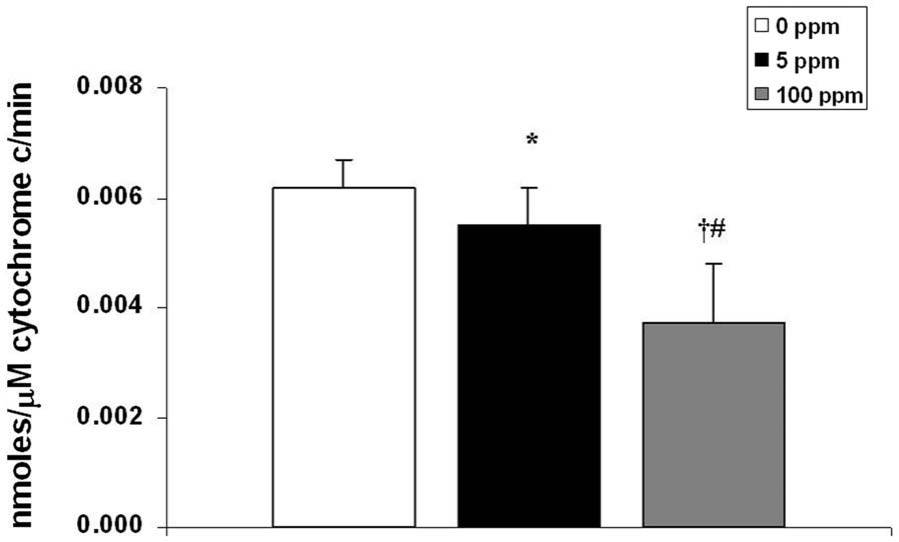
Cytochrome c peroxidase is inhibited following CO exposure. Steady-state cytochrome c peroxidase activity immediately following 3-hour exposure is shown. Values are expressed as means plus standard deviation. N = 5 animals per cohort. *P<.05 vs. 0 ppm. **†** *P*<.01 vs. 0 ppm. **#** *P*<.05 vs. 5 ppm.

Cytochrome c peroxidation of cardiolipin is an important step for cytochrome c release and initiation of the intrinsic apoptosis pathway [22]. Nanomolar concentrations of CO can bind to the cytochrome c-cardiolipin complex within mitochondria, inhibiting cytochrome c peroxidase activity [23]. Thus, immediately following 3-hour exposure to either 0 ppm CO (air), 5 ppm CO, or 100 ppm CO, we isolated mitochondria from 10 day old mouse pup brain, extracted cytochrome c, and measured peroxidase activity of cytochrome c using spectrophotometry. CO exposure significantly decreased the peroxidase activity of brain cytochrome c in a dose-dependent manner (figure 6). These findings suggest CO-mediated inhibition of cytochrome c peroxidase activity as a potential mechanism for impaired cytochrome c release.

### Brief CO exposure increases NeuN protein, neuron number, and results in megalencephaly

**Figure 7:**
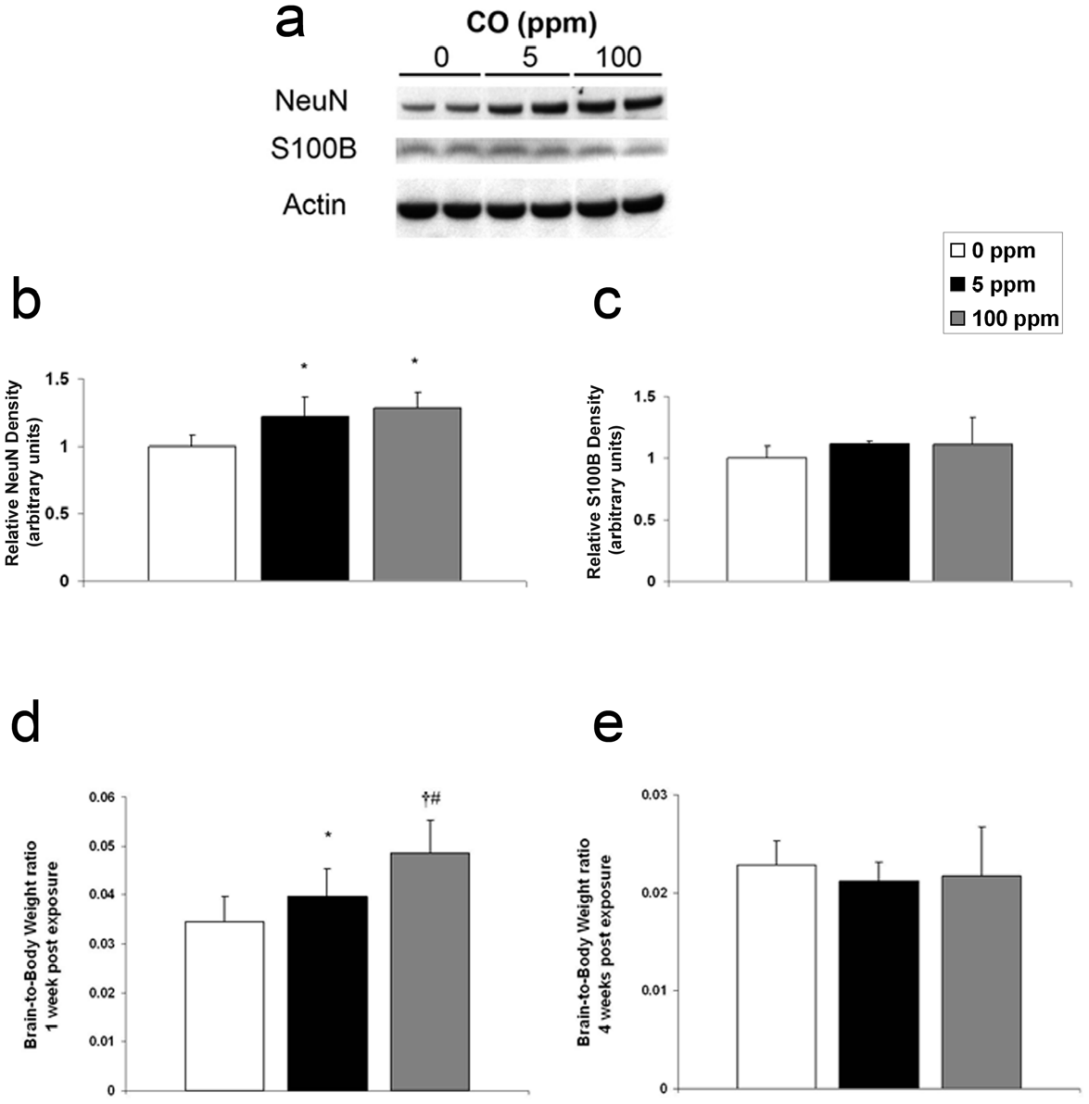
Neuron specific antigen and relative brain size increase following CO exposure. Cohorts were evaluated one week following 3-hour exposure to air (0 ppm CO), 5 ppm CO, or 100 ppm CO. (**a**) A representative immunoblot of neuron specific antigen (NeuN) and is depicted. CO exposed cohorts are demonstrated (0 ppm, 5 ppm, and 100 ppm). Actin was used as a loading control. Graphical representations of (**b**) NeuN and (**c**) S100β relative densities are shown. Air exposed values were arbitrarily set to 1. N = 5. Brain-to-body weight ratios (**d**) one week post exposure (N = 8 animals per cohort) and (**e**) four weeks post exposure (N = 10 animals per cohort) are demonstrated. Values are expressed as means plus standard deviation. *P<.05 vs. 0 ppm. **†** *P*<.001 vs. 0 ppm. **#** *P*<.01 vs. 5 ppm.

**Figure 8:**
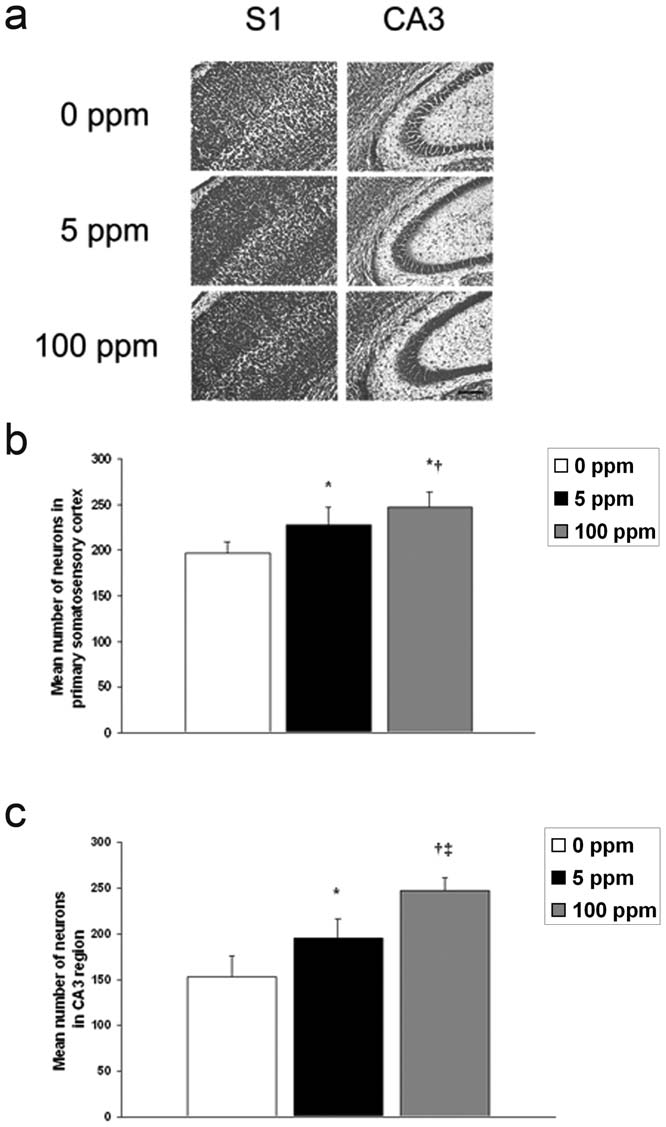
Number of neurons in neocortex and hippocampus increase following CO exposure. (**a**) Cresyl violet staining of coronal sections one week following 3-hour exposure to air (0 ppm CO), 5 ppm CO, or 100 ppm CO are demonstrated. S1 is primary somatosensory cortex; CA3 is CA3 region of hippocampus. Scale bar, 200 µm. (**b**) Quantification of the number of neurons in primary somatosensory cortex. Values are expressed as means plus standard deviation. *P<.001 vs. 0 ppm. †*P*<.03 vs. 5 ppm. (**c**) Quantification of the number of neurons in CA3 region of hippocampus. *P<.001 vs. 0 ppm. †*P*<.00001 vs. 0 ppm. ‡ *P*<.001 vs. 5 ppm. N = 3 animals per cohort.

The results indicate that the effects of sub-clinical CO exposure on developmental apoptosis are immediate and transient on PND 10 within the hippocampus but more prolonged within the neocortex. Thus, we hypothesized that CO-mediated inhibition of neuroapoptosis would decrease cell dropout and would subsequently result in an excess number of neurons and larger brains. To test this, we evaluated newborn mice one week following 3-hour exposure to either 0 ppm CO (air), 5 ppm CO, or 100 ppm CO. We determined steady-state levels of neuron specific antigen (NeuN) with immunoblotting, quantified the number of neurons using cresyl violet staining, and measured brain-to-body weight ratios. Brain NeuN significantly increased in both cohorts following CO exposure compared to air-exposed controls (figure 7). CO exposure, however, resulted in no change in S100β a calcium-binding protein localized predominantly in astroglial cells [24] (figure 7). The number of cells increased significantly in a dose dependent manner one week following CO exposure in the primary somatosensory cortex and in the CA3 region of the hippocampus suggesting a CO-mediated increase in the number of neurons (figure 8). In support of this, brain-to-body weight ratio increased significantly in a dose-dependent manner one week following CO exposure compared to controls (figure 7d). In a separate cohort, we assessed brain-to-body weight ratios 4 weeks after the 3-hour exposure. We found there to be no difference between groups at this later time point (figure 7e). Interestingly, brain weight one week post exposure to 100 ppm CO was equivalent to brain weight at the 4 week time point (data not shown). This suggests that relative megalencephaly following CO exposure was transient.

### Postnatal sub-clinical CO exposure impairs learning, memory, and social behavior

**Figure 9:**
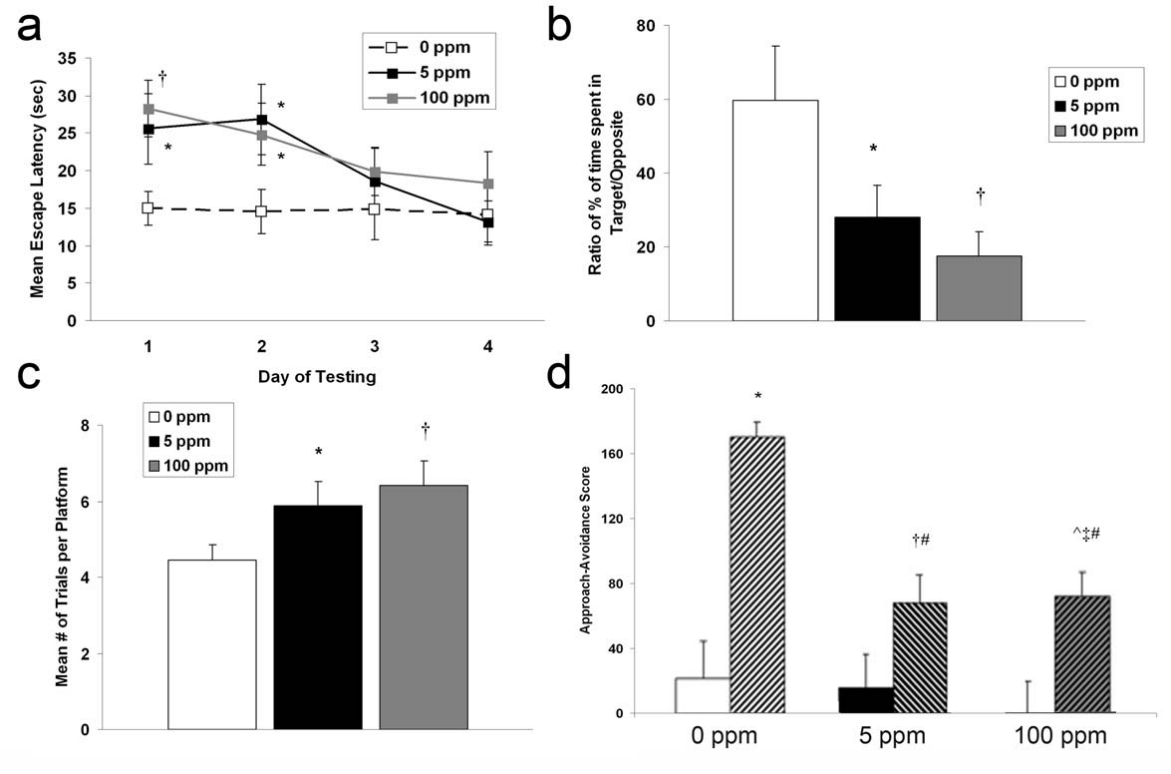
Memory, learning, and social behavior are impaired following CO exposure. (**a–c**) Morris water maze results are shown. (**a**) Escape latency time (in seconds) is demonstrated for the four days of reference memory testing. Values are expressed as means plus standard deviation. N = 13 animals per cohort. F value = 3.08. *P<.03 vs. 0 ppm. †*P*<.01 vs. 0 ppm. (**b**) Probe test is expressed as the ratio of percent time spent in the target quadrant versus the opposite quadrant. F value = 2.66. (**c**) Spatial working memory is expressed as the mean number of trials required to reach the hidden platform. Values are expressed as means plus standard deviation. N = 13 animals per cohort. F value = 2.73. *P<.05 vs. 0 ppm. †*P*<.01 vs. 0 ppm. (**d**) Social approach-avoidance scores for the CO-exposed cohorts are shown. Solid colored bars represent scores in absence of stimulus mouse (phase 1). Hashed bars represent scores in presence of stimulus mouse (phase 2). Values are expressed as means plus standard deviation. N = 13 animals per cohort. F value = 3.15. *P<.001 vs. 0 ppm phase 1. †*P*<.05 vs. 5 ppm phase 1. ‡*P*<.02 vs. 100 ppm phase 1. ∧ *P*<.05 vs. 5 ppm phase 1. #*P*<.001 vs. 0 ppm phase 2.

Megalencephaly is associated with neurodevelopmental dysfunction and is seen in a variety of behavior disorders [25]. Thus, we tested reference memory and spatial learning memory 4–5 weeks post exposure using Morris water maze behavioral assays. Escape latency was significantly longer on day 1 and 2 of testing in both CO exposed cohorts compared to controls indicating impaired reference memory (figure 9a). However, escape latency following CO exposure reached air-exposed control values on day 3 and 4 of testing suggesting that defects in reference memory could be overcome (figure 9a). Probe test following removal of the platform demonstrated that CO exposed mice spent significantly less time in the target quadrant indicating impaired memory retention (figure 9b). With a hidden platform and allowing visual cues, CO exposed mice required significantly more trials to reach the platform indicating impaired spatial working memory (figure 9c). These deficiencies in learning and memory appeared to be dose dependent.

Disturbances in socialization are common features of a variety of neurodevelopmental disorders. Thus, we assessed social behavior in a cohort of mice 4 weeks following brief postnatal exposure with social approach-avoidance testing. Using a 3-chambered behavioral apparatus, approach-avoidance scores were determined based on the frequency the test mouse approached or avoided the stimulus mouse [26]. Air-exposed controls showed a marked increase in approach-avoidance score when the stimulus mouse was placed in the chamber (figure 9d). This is indicative of intact socialization behavior. In contrast, both CO exposed cohorts demonstrated significantly lower approach-avoidance scores compared to controls indicating reduced social approach and greater avoidance (figure 9d). There was no difference between CO exposed groups in approach-avoidance with regard to the stimulus mouse (figure 9d).

## Discussion

**Figure 10:**
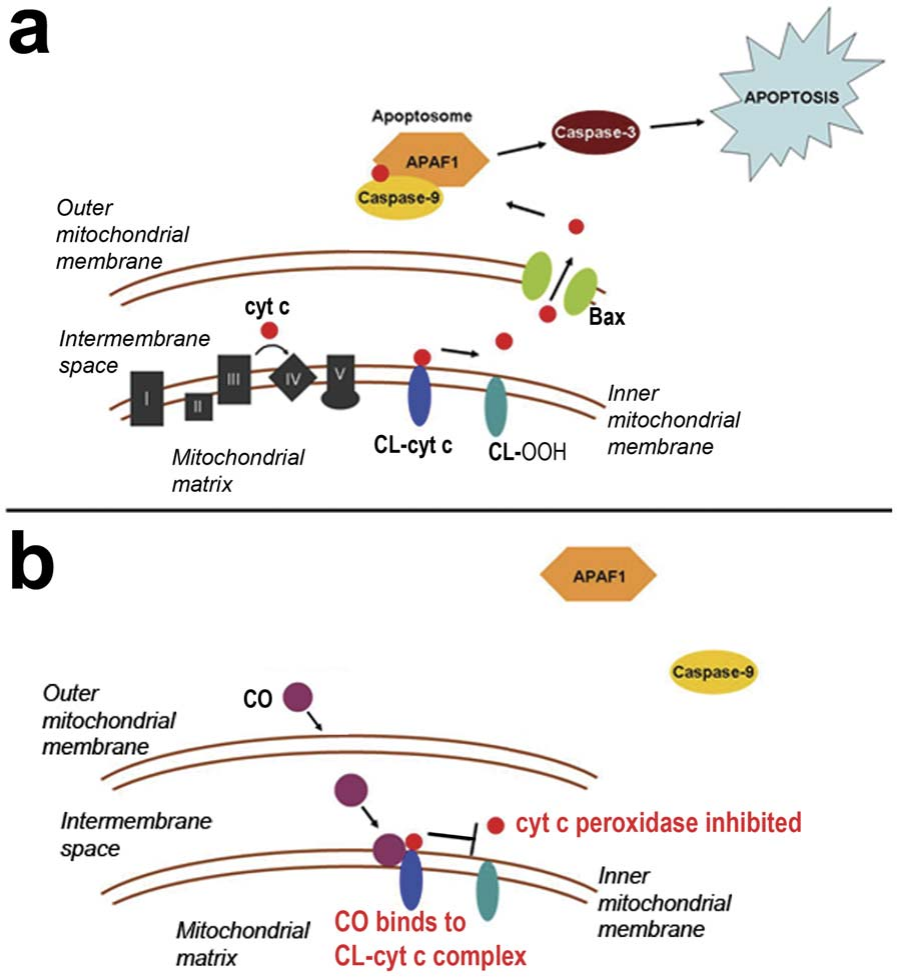
Mechanism of CO-mediated inhibition of apoptosis. (**a**) The intrinsic pathway of apoptosis is depicted. Cytochrome c (cyt c), the mobile electron carrier between Complexes III and IV in the electron transport chain, is bound to cardiolipin (CL) on the inner mitochondrial membrane via both electrostatic and hydrophobic interactions. Cyt c has peroxidase activity and, in the presence of hydrogen peroxide, oxidizes CL to hydroperoxycardiolipin (CL-OOH). This mobilizes cyt c from the inner membrane and permits cyt c release following permeabilization of the outer membrane. (**b**) Mechanism of carbon monoxide (CO) inhibition of cyt c peroxidase is shown. CO diffuses across the outer mitochondrial membrane, binds to the cyt c-CL complex, and inhibits the cyt c peroxidase activity. This prevents oxidation of CL, mobilization of cyt c, cyt c release, and subsequent caspase activation.

Here we demonstrate that 3-hour exposure to low concentrations of CO on PND 10 inhibits developmental neuroapoptosis. Our data suggest the underlying mechanism is inhibition of cytochrome c peroxidase activity resulting in impaired cytochrome c release from mitochondria. Timing and duration of CO-mediated inhibition of apoptosis was brain region dependent and was associated with excess neuronal protein, increased number of neurons, and megalencephaly. Furthermore, brief postnatal sub-clinical CO exposure was associated with long term defects in learning and memory and impaired social behavior. Although low concentrations of inhaled CO (50–500 ppm) have been previously shown to exert anti-apoptotic effects in endothelium, vascular smooth muscle, liver, and lung tissue during hyperoxia, sepsis, and ischemia-reperfusion, this is the first evaluation of the effect of brief sub-clinical CO exposure on apoptosis in the developing brain [27]–[31]. Our findings of CO-mediated inhibition of cytochrome c peroxidase activity are consistent with prior *in vitro* studies and such inhibition represents a plausible mechanism for CO-mediated impairment of cytochrome c release and interruption of the intrinsic apoptosis pathway [23]. Importantly, this is the first report of CO-mediated inhibition of cytochrome c peroxidase *in vivo*. Figure 10 details this anti-apoptotic mechanism.

Although the duration of CO exposure was only for 3 hours, the anti-apoptotic effects persisted for at least 72 hours in the neocortex and activated caspase-3 failed to return to PND 10 control levels in the hippocampus following CO exposure. Thus, the effects of a brief 3-hour CO exposure were prolonged. Because developmental apoptosis is a finite process and synaptogenesis is completed by the second or third week of life in rodents, inhibition of neuroapoptosis for several days during this critical period likely has profound consequences on brain development. Neuroapoptosis is critical and necessary for synaptogenesis, removal of aberrant neuronal connections, and may regulate synaptic plasticity during brain development [10]. Although not tested here, it is possible that CO-mediated inhibition of neuroapoptosis impaired neuron pruning and synapse elimination, resulting in an abundance of aberrant neuronal connections. Such an effect could explain the CO-induced increases in NeuN content, neuronal number, and brain size as well as the defects in learning, memory, and impaired social behavior that we have described.

Prolonged CO-mediated inhibition of apoptosis following brief exposure may be attributed to the amount of COHb formed. Although COHb levels returned toward PND 10 control values by 24 hours following CO exposure, they remained mildly but significantly elevated compared to time-matched controls even up to 72 hours post exposure. Thus, tissue unloading of CO from hemoglobin, as dictated by the Haldane equation, persisted during this time period and brain exposure to CO likely continued for at least 72 hours following the acute exposure [32]. Finding significantly decreased numbers of activated caspase-3 cells in the neocortex 24 and 72 hours post CO exposure supports this notion.

The half-life of COHb in human adults is 240 to 320 minutes while breathing room air [1]. Because mice do not express fetal hemoglobin, our data indicate the consequences and timeline of a brief CO exposure in the context of adult-type hemoglobin [33]. Fetal hemoglobin, on the other hand, has a much higher affinity for CO than adult hemoglobin, making the elimination half-life longer in newborns and infants [34]. With up to 80% of circulating hemoglobin being fetal-type hemoglobin in full term human newborns, elevated COHb levels would likely persist even longer following a similar short-term CO exposure and result in even more prolonged CO delivery to the brain [35]. Thus, it is entirely possible that a single CO exposure lasting several hours or multiple CO exposures during infancy could result in prolonged brain exposure to CO. Our data suggest that this could have neurodevelopmental consequences.

Defects in programmed cell death in the developing brain have been shown to have profound effects. Knockout of caspase-3 or caspase-9, for example, results in severe structural abnormalities in the postnatal mouse brain [36], [37]. The morphologic consequences of caspase-3 and -9 deficiencies include hyperplasia of neurons in the cortex, cerebellum, striatum, hippocampus, and retina as well as neuronal disorganization [36], [37]. Finding increased brain NeuN content, greater number of neurons, and relatively larger brains following CO exposure suggests impaired neuron elimination and is consistent with the known effects of disrupted programmed cell death in the developing brain [36], [37].

Abnormalities in brain size and growth are known characteristics of a variety of inherited syndromes such as Sotos syndrome, Fragile X syndrome (autism spectrum), and Simpson-Golabi-Behmel syndrome [25]. With the autism spectrum, for example, brain enlargement begins within the first few postnatal years, resulting in increased brain weight and volume by 2 to 4 years of age followed by an arrested growth period and possible decline in brain size [38], [39]. The megalencephalic brain in autism has also been shown to have significantly more neurons than unaffected children [38]. It is important to note that megalencephaly is not specific for autism and not a requisite for diagnosis [40]. However, the temporal changes in brain size and increased number of neurons we have demonstrated following CO exposure in mice are reminiscent of features that have been reported in autism [38], [39]. Although the exact cause or causes of autism and autism spectrum disorders are unknown, current hypotheses point toward environmental insults and genetic predisposition [41]. Residing in an urban setting has been identified as a risk factor, yet CO has never been evaluated as a potential cause of autism [42]. Our findings suggest that future investigation should explore CO as a potential environmental contributor to the development of the autism spectrum.

If the neurodevelopmental effects of postnatal sub-clinical CO exposure described here translate to the human state, then these findings could have profound public health implications. This is because low levels of CO are commonly encountered in a variety of indoor and outdoor settings. Annual outdoor CO levels have averaged between 2 and 5 ppm since the year 2000 in a number of US cities and short-term peaks occur each day and vary based on season (http://www.epa.gov/airquality/carbonmonoxide/). CO levels can reach between 10 and 12 ppm within vehicles during heavy traffic and even higher concentrations are encountered in semi-closed environments exposed to vehicle exhaust, such as parking garages and tunnels (http://www.epa.gov/airquality/carbonmonoxide/). Indoor levels can rise to 100 ppm with use of a gas stove and it has been shown that students exposed to between 17 and 100 ppm CO generated by kerosene stoves for up to two and a half hours have defects in memory, learning, concentration, abstract thinking, and visual-motor skills [1], [43]. Thus, infants may be exposed to low concentrations of CO in a variety of different environments and settings and finding that 5 ppm CO has neurodevelopmental consequences is relevant and important.

Another important source of CO is tobacco smoke [44]. Cigarette smoke contains almost 5000 chemical compounds, including CO, and approximately 10 million children under the age of 6 years are exposed to second hand tobacco smoke in the homes of caregivers and relatives [44]. CO levels can range between 5 and 35 ppm within smoking rooms and can vary based on the number of lit cigarettes and the size of the room [45], [46]. Much of the work evaluating the neurotoxic effects of smoking on the developing brain has focused on prenatal exposure and has demonstrated an association between smoking during pregnancy and subsequent neurocognitive impairments and behavioral abnormalities in their children [44], [47], [48]. In a recent study of more than 55,000 US children younger than 12 years of age, second hand smoke exposure was shown to increase the odds of having 2 or more neurobehavioral disorders by 50% compared to those not exposed [49]. In a study of over 2000 US children between the ages of 4 to 11 years, postnatal exposure to maternal smoking was found to be associated with a significantly higher incidence of behavioral problems, even after controlling for confounders [47]. Importantly, this work identified a dose-response relationship between the amount of postnatal smoke exposure and manifestation of behavior disorders [47]. Such a dose-effect response has also been shown with prenatal maternal smoking [50]. In our work, we identified dose dependent consequences of CO exposure with regard to cytochrome c peroxidase activity, neuroapoptosis, neuron number, relative brain size, and impaired learning and memory behavior. Thus, it is quite possible that CO causes or contributes to the acquired neurodevelopmental abnormalities in children who are passively exposed to tobacco smoke.

It was previously thought that short-term exposures to low concentrations of CO were innocuous and that the absence of symptoms during exposure indicated that such exposures were not harmful. However, our data suggest that brief sub-clinical CO exposure during a critical time in development could have profound and long lasting neurodevelopmental implications. Although much of our data suggest a dose-dependent response based on CO concentration, we have only evaluated the effects of exposure to two different concentrations of CO for a specific duration of time in mice of a specific age. There are likely vulnerabilities and differential effects based on age at time of exposure, CO concentration exposed to, and a critical time-weighted duration of exposure. If such exposures during infancy and childhood are shown to result in neurocognitive and behavioral deficits in humans, then we will need to be much more knowledgeable about the air we breathe and the environments we expose our children to. It is likely that new safety standards will need to be considered and efforts will need to focus on further reducing environmental CO and preventing childhood exposures.

## Materials and Methods

### CO exposure

The care of the animals in this study was in accordance with NIH and Institutional Animal Care and Use Committee guidelines. Study approval was granted by the Children's National Medical Center and University of Pennsylvania IACUC (protocol # 236-08-20). Six to eight week old CD-1 pregnant female mice (20–30 grams) were acquired (Charles River, Wilmington MA) to yield newborn pups. CD-1 mice were chosen because pups have been shown to reliably demonstrate neuronal changes consistent with human neonatal hypoxic injury in experimental models aimed to induce such an injury [51]. On postnatal day 10 (PND 10), we exposed CD-1 mouse pups to 0 ppm CO (air), 5 ppm CO in air, or 100 ppm CO in air for 3 hours in a 7-liter Plexiglas chamber (25 cm×20 cm×14 cm). The three experimental cohorts represented: negative control (0 ppm CO), low concentration sub-clinical CO (5 ppm), high concentration sub-clinical CO (100 ppm). The chamber had a port for fresh gas inlet and a port for gas outlet which was directed to a fume hood exhaust using standard suction tubing. Specific concentrations of CO in air (premixed gas H-cylinders, Air Products, Camden, NJ) were verified using an electrochemical sensing CO detector (Monoxor III, Bacharach, Anderson, CA). Designated CO mixtures were delivered through the chamber at a flow rate of 8–12 l/min. Mice were kept warm with heat lamps. Postnatal day 10 (PND 10) was chosen because synaptogenesis peaks at day 7 in rodents and is completed by the second or third week of life [11], [14]. Thus, PND 10 in the mouse is probably equivalent to the postnatal human infant [15]–[17]. Three hour exposure time was chosen to represent a brief, short-term exposure. Following exposure, pups were placed with their respective dams. Pups were shown to maintain blood glucose levels following the 3 hour exposure and did not become hypoglycemic (data not shown). A total of 348 newborn mice were evaluated (equal numbers of male and female [174 each]).

### Carboxyhemoglobin (COHb) levels

COHb levels were measured immediately following 3-hour exposure, 24 hours post exposure, and 72 hours post exposure in separate cohorts. At the time of euthanasia, following pentobarbital injection (150 mg/kg, ip), 200 µL of blood was sampled from the left ventricle and COHb measured via 6 wavelength co-oximetry (Radiometer Osm3 Hemoximeter, Copenhagen, Denmark, range 0–100±0.2%). Eight animals per cohort per time point were evaluated.

### Activated caspase-3 immunohistochemistry

Pups were evaluated 2, 24, or 72 hours after 3-hour exposure. At the time of euthanasia, following pentobarbital injection (150 mg/kg, ip), the brain was perfused with 4% paraformaldehyde in 0.1 M phosphate buffer (pH 7.4) via left ventricle injection for 30 min and then post-fixed in additional fixative solution for 24 h at 4°C. Serial frozen sections were cut at a thickness of 6-µm in the coronal plane through the cerebral hemispheres beginning at −1.7 mm from bregma, 2.1 mm from interaural and individual sections were slide mounted. Immunohistochemistry was performed on three to four non-serial non-adjacent sections using polyclonal anti-rabbit activated caspase-3 (Cell Signaling Technology, Beverly, MA, 9661) at a concentration of 1∶1500, biotinylated secondary antibody (goat anti-rabbit, Cell Signaling Technology) at a concentration of 1∶200, and developed with DAB. The number of activated caspase-3 positive cells per mm^2^ was quantified at 10× magnification in neocortex and hippocampus of both hemispheres in 3 to 4 animals per group per time point (2 males, 2 females in 0 ppm cohort; 2 males, 1 female in both CO exposed cohorts).

### Caspase-3,7 activity

Following euthanasia with pentobarbital 2 hours post CO exposure, brains were immediately harvested and placed on ice. Cytosol from whole brain was isolated with differential centrifugation, and caspase-3,7 activity was measured using a fluorometric immunosorbent enzyme assay in a medium containing 0.32 M sucrose, 10 mM Tris–HCl buffer (pH 7.0), 3 mM MgCl_2_, 500 µg cytosolic protein, and 75 µM Ac-Asp-Glu-Val-Asp-amino-4-methyl-coumarin, a fluorogenic substrate for caspase-3 (Alexis Biochemicals, San Diego, CA). Activity was determined spectrofluorometrically at 460 nm using an excitation wavelength of 380 nm at 37°C continuously for 300 seconds. The rate of reaction was measured and activity calculated using amino-4-methyl-coumarin as a standard. Caspase-3,7 activity of air-exposed mice was arbitrarily set to equal 1. Ten animals per group were evaluated.

### Terminal deoxynucleotidyl transferase (TdT)-mediated UTP nick end-labeling (TUNEL) staining

Pups were evaluated 2 hours post 3-hour exposure. At the time of euthanasia, the brain was perfused with 4% paraformaldehyde in 0.1 M phosphate buffer (pH 7.4) via left ventricle injection for 30 min and then post-fixed in additional fixative solution for 24 h at 4°C. Paraffin embedded brain sections were cut into 6-µm sections in the coronal plane through the cerebral hemispheres beginning at −1.7 mm from bregma, 2.1 mm from interaural, slide mounted, and stained for TUNEL. Sections were incubated in 0.5% Triton at room temperature, followed by proteinase K at 37°C, then immersed in TdT buffer (30 mmol/L Tris-HCl buffer, pH 7.2, 140 mmol/L sodium cacodylate, and 1 mmol/L cobalt chloride) at room temperature. This was followed by incubation with TdT and biotin-16-dUTP for 60 minutes at 37°C. The reaction was terminated with TB buffer (300 mmol/L sodium chloride with 30 mmol/L sodium citrate) at room temperature, followed by immersion in 3% hydrogen peroxide and 2% FBS at room temperature. The sections were then covered with an Avidin Biotin Complex (1∶200 dilution) for 30 minutes at room temperature, incubated with FITC-Avidin D for detection and counterstained with DAPI. The numbers of TUNEL positive nuclei in neocortex and hippocampus were quantified in 3 to 4 non-serial sections per mouse and 3 to 4 mice per cohort were evaluated (2 males, 2 females in 0 ppm cohort; 2 males, 1 female in both CO exposed cohorts).

### Heme c determination

Brain mitochondria and cytosol were isolated by differential centrifugation [52]. As previously described, whole brain was harvested and homogenized in ice-cold H medium (70 mM sucrose, 220 mM mannitol, 2.5 mM Hepes, pH 7.4 and 2 mM EDTA) [52]. The homogenate was spun at 1500× *g* for 10 min at 4°C. Supernatant was removed and centrifuged at 10,000× *g* for 10 min at 4°C. Cytosolic supernatant was collected and pellet was resuspended in H medium and centrifuged again at 10,000× *g* for 10 min at 4°C. Pellet was again resuspended in H medium and mitochondrial and cytosolic protein concentrations subsequently determined using the method of Lowry [52].

Mitochondrial and cytosolic heme c content were calculated from the difference in spectra (dithionate/ascorbate reduced minus air-oxidized) of mitochondria or cytosolic protein (0.5–1 mg) solubilized in 10% lauryl maltoside using an absorption coefficient of 20.5 mM^−1^ cm^−1^ at 550 to 535 nm [53], [54]. Eight animals per cohort were evaluated.

### Cytochrome c peroxidase activity

Cytochrome c was extracted from fresh mitochondria as previously described [55]. Isolated brain mitochondria (20 mg/mL) were suspended in a hypotonic 0.015 M KCl solution for 10 min on ice and then centrifuged at 105,000× *g* for 15 min at 4°C. The pellet was resuspended in 0.15 M KCl solution for 10 min on ice and then centrifuged again at 105,000× *g* for 15 min at 4°C. The supernatant was collected and cytochrome c content quantified with spectrophotometry. The peroxidase activity of 0.5–1 µM cytochrome c was determined by measuring the rate of oxidation of 50 µM 2,2′-azinobis-(2-ethylbenzthiazoline-6-sulfonate) (ABTS) in 10 mM potassium phosphate buffer (pH 7.4) at 415 nm (ε415 = 3.6×104 M^−1^ cm^−1^) following the addition of hydrogen peroxide [56]. Five animals per cohort were evalulated.

### Immunoblot analysis

10 µg samples of homogenized brain protein were subjected to SDS-acrylamide gel electrophoresis and immunoblotting. Blots were labeled with a primary polyclonal antibody to bovine NeuN (Molecular Probes, Eugene, Oregon, USA) at a concentration of 1∶1000 and a primary monoclonal antibody to rabbit S100β (Millipore, Temecula, California) at a concentration of 1∶1000 and secondarily exposed to goat anti-bovine IgG (1∶2500) and donkey anti-rabbit IgG (1∶2500), respectively (Santa Cruz Biotechnology Inc., Santa Cruz, California). Signal was detected with enhanced chemiluminescence (ECL; Amersham Pharmacia Biotech, Piscataway, New Jersey, USA), and density was measured using scanning densitometry. Five animals per cohort were evaluated.

### Quantification of neuronal numbers

Pups were evaluated one week after 3-hour exposure. At the time of euthanasia, following pentobarbital injection (150 mg/kg, ip), the brain was perfused with 4% paraformaldehyde in 0.1 M phosphate buffer (pH 7.4) via left ventricle injection for 30 min and then post-fixed in additional fixative solution for 24 h at 4°C. Brains were stored in 30% sucrose for 24 hrs at 4°C and sectioned coronally (45 µm) on a freezing microtome. Sections were rinsed in PBS, slide mounted and stained with cresyl violet for 30 min [57]. Cresyl violet-positive neurons with a clear nucleus and nucleoli in the primary somatosensory cortex and in the pyramidal layer of the CA3 region of the hippocampus were counted by two blinded observers using an image analyzing system equipped with a computer-based CCD camera (Nikon Eclipse e800). Starting from the first section (interaural 2.10 mm, bregma −1.70 mm, 4.7 mm from the most rostral section), counts were taken from 5 coronal sections at 0.135 mm increments in 100,000 µm^2^ fields in both hemispheres per mouse [58]. Brain regions were defined in accordance with Mouse Brain Atlas [59], [60]. Three mice per cohort were evaluated.

### Brain-to-body weight ratio

Separate cohorts of mice were evaluated 1 week post exposure (N = 8) or 4 weeks post exposure (N = 10). Following euthanasia with pentobarbital (150 mg/kg, ip), body weight was measured with a calibrated electronic scale. Fresh whole brain was dissected, dura removed, and medulla severed just distal to the cerebellum. Brain weight was measured with an analytical balance. Brain-to-body weight ratios were calculated.

### The Morris water maze

Mice were assessed 4–5 weeks post exposure. Water was maintained at 25–29°C and opacified with titanium (IV) dioxide. The mice were first trained to locate the 15 cm diameter submerged and flagged platform in the 1.5 m diameter pool. A white curtain surrounded the pool to minimize visual cues. Starting points were varied and mice were allotted 60 s to find the platform, or were guided to the platform, and remained on the platform for 10–15 s. The training was conducted over 2 days with 4 trails per day. *Reference memory:* Two days after cued training, the white curtain and platform flag were removed and fixed external visual cues were placed around the room. Starting points were varied and mice were allotted 60 s to find the hidden platform and the escape latency was recorded. The mice were tested for 4 days with 4 trials per day. *Probe test:* After the last trial on the last day of the reference memory testing, the platform was removed and the swim path data recorded for one 60 s trial. The starting location was in the “opposite” quadrant. The percent time spent in each quadrant was measured. *Spatial working memory*: Two days later, from varied starting points, mice were given 60 s to find the hidden platform in new pre-set locations. Visual cues in the room were required to locate the platform in less than 20 s for three consecutive trials and allowed up to 8 trials per day for 10 days. A new platform location was selected the day after criterion was reached. The number of trials to reach criterion for each platform were recorded. Throughout all testing, the mice were actively heated after each trial with heat lamps and the interval between trials was 30–45 min. A video camera mounted above the pool tracked and recorded the swim paths of all mice with a computer running IMAQ PCI-1407 software [61]. N = 13 animals per group.

### Social approach-avoidance assay

A separate cohort of mice was assessed 4–5 weeks post exposure. The assay was conducted in a 3-chambered black plexiglass apparatus [26]. Two identical clear plexiglass cylinders allowing ventilation were installed in the two end chambers. Social investigation between either a stimulus mouse or an inanimate object inside the cylinder was tested. The assay included two phases. In phase 1, the test mouse freely explored the chamber for 10 min for habituation. In phase 2, a stimulus mouse was introduced into one cylinder and the inanimate object (a 3×2 cm white pvc tube) into the other cylinder for 10 min. The stimulus mice were 3–4 week old gonadectomized A/J mice. For each test, a stimulus and test mouse of same sex were paired. To facilitate and standardize the scoring, we used a computer-assisted scoring method: Image J (from the NIH, http://rsb.info.nih.gov/ij/). Videos were converted to uncompressed AVI files and then analyzed using Image J. The ROI was defined. After thresholding, the percent time spent in each chamber was calculated by dividing the number of frames that contained signal (the presence of test mouse) by the total number of frames for each phase and multiplying by 100. N = 13 animals per group.

### Statistics

Data are presented as mean ± standard deviation. Statistical significance was assessed using one-way ANOVA and post hoc Tukey's test with *P*<.05. For MWM we used 2-way ANOVA with repeated measures and Bonferroni posttests (Reference Memory), unpaired two-tailed t-test (Probe Test, Spatial Memory). For the sociability behavioral analyses, the “approach-avoidance score” was calculated. The scores were analyzed with a 3×2-way ANOVA (CO exposure condition×phase 1/2) with repeated measures on phases 1 and 2. Analyses were performed using GraphPad Prism 5.03 software.

## References

[1] Kao LW, Nañagas KA (2004). Carbon monoxide poisoning.. Emerg Med Clin North Am.

[2] Smithline HA, Ward KR, Chiulli DA, Blake HC, Rivers EP (2003). Whole body oxygen consumption and critical oxygen delivery in response to prolonged and severe carbon monoxide poisoning.. Resuscitation.

[3] Hauck H, Neuberger M (1984). Carbon monoxide uptake and the resulting carboxyhemoglobin in man.. Eur J Appl Physiol Occup Physiol.

[4] Gorman D, Drewry A, Huang YL, Sames C (2003). The clinical toxicology of carbon monoxide.. Toxicology.

[5] Kao LW, Nanagas KA (2005). Carbon monoxide poisoning.. Med Clin North Am.

[6] Winter PM, Miller JN (1976). Carbon monoxide poisoning.. JAMA.

[7] Gomez C, Berlin I, Marquis P, Delcroix M (2005). Expired air carbon monoxide concentration in mothers and their spouses above 5 ppm is associated with decreased fetal growth.. Prev Med.

[8] Di Giovanni V, Cagiano R, De Salvia MA, Giustino A, Lacomba C (1993). Neurobehavioral changes produced in rats by prenatal exposure to carbon monoxide.. Brain Res.

[9] Webber DS, Korsak RA, Sininger LK, Sampogna SL, Edmond J (2003). Mild carbon monoxide exposure impairs the developing auditory system of the rat.. J Neurosci Res.

[10] Chan WY, Lorke DE, Tiu SC, Yew DT (2002). Proliferation and apoptosis in the developing human neocortex.. Anat Rec.

[11] Rice D, Barone S (2000). Critical periods of vulnerability for the developing nervous system: evidence from humans and animal models.. Environ Health Perspect.

[12] Blomgren K, Leist M, Groc L (2007). Pathological apoptosis in the developing brain.. Apoptosis.

[13] Jiang Y, de Bruin A, Caldas H, Fangusaro J, Hayes J (2005). Essential role for survivin in early brain development.. J Neurosci.

[14] Sanno H, Shen X, Kuru N, Bormuth I, Bobsin K (2010). Control of postnatal apoptosis in the neocortex by RhoA-subfamily GTPases determines neuronal density.. J Neurosci.

[15] Klintsova AY, Helfer JL, Calizo LH, Dong WK, Goodlett CR (2007). Persistent impairment of hippocampal neurogenesis in young adult rats following early postnatal alcohol exposure.. Alcohol Clin Exp Res.

[16] Clancy B, Finlay BL, Darlington RB, Anand KJ (2007). Extrapolating brain development from experimental species to humans.. Neurotoxicology.

[17] Hornig M, Chian D, Lipkin WI (2004). Neurotoxic effects of postnatal thimerosal are mouse strain dependent.. Mol Psychiatry.

[18] Nasr V, Emmanuel J, Deutsch N, Slack M, Kanter J (2010). Carbon monoxide re-breathing during low-flow anaesthesia in infants and children.. Br J Anaesth.

[19] Ferrer I, Bernet E, Soriano E, del Rio T, Fonseca M (1990). Naturally occurring cell death in the cerebral cortex of the rat and removal of dead cells by transitory phagocytes.. Neuroscience.

[20] Ferrer I, Serrano T, Soriano E (1990). Naturally occurring cell death in the subicular complex and hippocampus in the rat during development.. Neurosci Res.

[21] Kim WR, Sun W (2011). Programmed cell death during postnatal development of the rodent nervous system.. Dev Growth Differ.

[22] Kagan VE, Tyurin VA, Jiang J, Tyurina YY, Ritov VB (2005). Cytochrome c acts as a cardiolipin oxygenase required for release of proapoptotic factors.. Nat Chem Biol.

[23] Kapetanaki SM, Silkstone G, Husu I, Liebl U, Wilson MT (2009). Interaction of carbon monoxide with the apoptosis-inducing cytochrome c-cardiolipin complex.. Biochemistry.

[24] Berger RP, Adelson PD, Pierce MC, Dulani T, Cassidy LD (2005). Serum neuron-specific enolase, S100B, and myelin basic protein concentrations after inflicted and noninflicted traumatic brain injury in children.. J Neurosurg.

[25] Olney AH (2007). Macrocephaly syndromes.. Semin Pediatr Neurol.

[26] Brodkin ES, Hagemann A, Nemetski SM, Silver LM (2004). Social approach-avoidance behavior of inbred mouse strains towards DBA/2 mice.. Brain Res.

[27] Zhou H, Liu J, Pan P, Jin D, Ding W (2010). Carbon monoxide inhalation decreased lung injury via anti-inflammatory and anti-apoptotic effects in brain death rats.. Exp Biol Med.

[28] Song R, Kubo M, Morse D, Zhou Z, Zhang X (2003). Carbon monoxide induces cytoprotection in rat orthotopic lung transplantation via anti-inflammatory and anti-apoptotic effects.. Am J Pathol.

[29] Bernardini C, Zannoni A, Bacci ML, Forni M (2010). Protective effect of carbon monoxide pre-conditioning on LPS-induced endothelial cell stress.. Cell Stress Chaperones.

[30] Sarady JK, Zuckerbraun BS, Bilban M, Wagner O, Usheva A (2004). Carbon monoxide protection against endotoxic shock involves reciprocal effects on iNOS in the lung and liver.. FASEB J.

[31] Wang X, Wang Y, Kim HP, Nakahira K, Ryter SW (2007). Carbon monoxide protects against hyperoxia-induced endothelial cell apoptosis by inhibiting reactive oxygen species formation.. J Biol Chem.

[32] Jain KK, Jain KK, Textbook of Hyperbaric Medicine (2009). Carbon Monoxide and Other Tissue Poisons..

[33] McConnell SC, Huo Y, Liu S, Ryan TM (2011). Human globin knock-in mice complete fetal-to-adult hemoglobin switching in postnatal development.. Mol Cell Biol.

[34] Berman S (2003). Pediatric Decision Making: Carbon Monoxide Poisoning.

[35] Proytcheva MA (2009). Issues in neonatal cellular analysis.. Am J Clin Pathol.

[36] Kuida K, Zheng TS, Na S, Kuan C, Yang D (1996). Decreased apoptosis in the brain and premature lethality in CPP32-deficient mice.. Nature.

[37] Hakem R, Hakem A, Duncan GS, Henderson JT, Woo M (1998). Differential requirement for caspase 9 in apoptotic pathways in vivo.. Cell.

[38] Courchesne E, Campbell K, Solso S (2011). Brain growth across the life span in autism: age-specific changes in anatomical pathology.. Brain Res.

[39] Schumann CM, Bloss CS, Barnes CC, Wideman GM, Carper RA (2010). Longitudinal magnetic resonance imaging study of cortical development through early childhood in autism.. J Neurosci.

[40] Barnard-Brak L, Sulak T, Hatz JK (2011). Macrocephaly in children with autism spectrum disorders.. Pediatr Neurol.

[41] Herbert MR (2010). Contributions of the environment and environmentally vulnerable physiology to autism spectrum disorders.. Curr Opin Neurol.

[42] Rosenberg RE, Daniels AM, Law JK, Law PA, Kaufmann WE (2009). Trends in autism spectrum disorder diagnoses: 1994–2007.. J Autism Dev Disord.

[43] Amitai Y, Zlotogorski Z, Golan-Katzav V, Wexler A, Gross D (1998). Neuropsychological impairment from acute low-level exposure to carbon monoxide.. Arch Neurol.

[44] Rauh VA, Horton MK, Miller RL, Whyatt RM, Perera F (2010). Neonatology and the Environment: Impact of Early Exposure to Airborne Environmental Toxicants on Infant and Child Neurodevelopment.. Neoreviews.

[45] Gül I, Karapinar H, Yarlioglues M, Ozdogru I, Kaya MG (2011). Acute effects of passive smoking on endothelial function.. Angiology.

[46] Jo WK, Oh JW, Dong JI (2004). Evaluation of exposure to carbon monoxide associated with passive smoking.. Environ Res.

[47] Weitzman M, Gortmaker S, Sobol A (1992). Maternal smoking and behavior problems of children.. Pediatrics.

[48] Eskenazi B, Castorina R (1999). Association of prenatal maternal or postnatal child environmental tobacco smoke exposure and neurodevelopmental and behavioral problems in children.. Environ Health Perspect.

[49] Kabir Z, Connolly GN, Alpert HR (2011). Secondhand smoke exposure and neurobehavioral disorders among children in the United States.. Pediatrics.

[50] Fergusson D, Woodward L, Horwood L (1998). Maternal smoking during pregnancy and psychiatric adjustment in late adolescence.. Arch Gen Psychiatry.

[51] Farahani R, Kanaan A, Gavrialov O, Brunnert S, Douglas RM (2008). Differential effects of chronic intermittent and chronic constant hypoxia on postnatal growth and development.. Pediatr Pulmonol.

[52] Piel DA, Gruber PJ, Weinheimer CJ, Courtois MR, Robertson CM (2007). Mitochondrial resuscitation with exogenous cytochrome c in the septic heart.. Crit Care Med.

[53] Ozawa T, Tanaka M, Shimomura Y (1980). Crystallization of the middle part of the mitochondrial electron transfer chain: Cytochrome bc1-cytochrome c complex.. Proc Natl Acad Sci USA.

[54] Tanaka M, Ogawa N, Ihara K, Sugiyama Y, Mukohata Y (2002). Cytochrome aa(3) in Haloferax volcanii.. J Bacteriol.

[55] Jacobs EE, Sanadi DR (1960). The reversible removal of cytochrome c from mitochondria.. J Biol Chem.

[56] Kim NH, Jeong MS, Choi SY, Kang JH (2004). Peroxidase Activity of Cytochrome c.. Bull Korean Chem Soc.

[57] Stenqvist A, Agerman K, Marmigère F, Minichiello L, Ernfors P (2005). Genetic evidence for selective neurotrophin 3 signalling through TrkC but not TrkB in vivo.. EMBO Rep.

[58] Kwon MS, Lee JK, Park SH, Sim YB, Jung JS (2010). Neuroprotective Effect of Visnagin on Kainic Acid-induced Neuronal Cell Death in the Mice Hippocampus.. Korean J Physiol Pharmacol.

[59] Franklin KBJ, Paxinos G (1997). The mouse brain in stereotaxic coordinates.

[60] Paxinos G, Watson C (2007). Atlas of the developing mouse brain: at E17.5, PO, and P6.

[61] Bianchi SL, Tran T, Liu C, Lin S, Li Y (2008). Brain and behavior changes in 12-month-old Tg2576 and nontransgenic mice exposed to anesthetics.. Neurobiol Aging.

